# Reporting the Impact of Pelvicalyceal System (PCS) Anatomy on Clinical Outcomes in Retrograde Intrarenal Surgery (RIRS) Studies: Can We Do Better? – Methodological Review from the Section of EAU Endourology

**DOI:** 10.5152/tud.2025.25032

**Published:** 2025-05-21

**Authors:** Ali Talyshinskii, Yerkebulan Mukhambetov, Ulanbek Zhanbyrbekuly, Lazaros Tzelves, Patrick Juliebø-Jones, Theodoros Tokas, Giorgio Bozzini, Wissam Kamal, Bhaskar Kumar Somani

**Affiliations:** 1 Department of Urology and Andrology, Astana Medical University, Astana, Kazakhstan; 2 Department of Urology and Oncology, Fergana Medical Institute of Public Health, Fergana, Uzbekistan; 3 Section of EAU Endourology Research Group; 4 Department of Urology, Sismanoglio Hospital, National and Kapodistrian University of Athens, Athens, Greece; 5 Department of Urology, Haukeland University Hospital, Bergen, Norway; 6 Department of Urology, University General Hospital of Heraklion, University of Crete School of Medicine, Heraklion, Greece; 7 Section of EAU Focal Therapy and Laparoscopy Group; 8 Department of Urology, Azienda Socio Sanitaria Territoriale Lariana, Como, Italy; 9 Section of EAU Endourology Bladder group; 10 Department of Urology, King Fahad General Hospital, Jeddah, Saudi Arabia; 11 Department of Urology, University Hospital Southampton NHS Trust, Southampton, UK

**Keywords:** RIRS, collecting system, PCS, clinical trial, methodology

## Abstract

To analyze available randomized clinical trials (RCTs) comparing retrograde intrarenal surgery (RIRS) with other modalities for urinary stone treatment to determine the extent of comparing the pelvicalyceal system (PCS) anatomy between patients. In December 2024, a search was conducted in databases and was limited to publications that describe comparisons of experimental and control groups in the context of RIRS for stones only in the kidney (PCS). Only RCTs comparing RIRS with other modalities without publication date restriction were included due to their highest level of evidence in the hierarchy of primary research. The parameters used in the selected studies were analyzed to compare the differences between the groups, focusing on PCS anatomy. The final analysis included 27 publications from 2421 articles. The presence and/or degree of hydronephrosis were analyzed in 8 studies. Direct morphometric measurements were compared in 4 studies and were focused on the lower pole only, namely the infundibulopelvic angle, infundibular length, and infundibular width. Features such as the position of the renal pelvis in relation to the kidney parenchyma (intrarenal, extrarenal), number and orientation of calyces, as well as the existing PCS classifications were not compared or used. This review shows gaps in the literature while assessing and reporting on PCS anatomy in studies with RIRS. Unless studies mention these anatomical factors without excluding certain groups of patients, it is difficult to compare outcomes between modalities and in between studies.

**Main Points**

- Pelvicalyceal system (PCS) represents a non-modifiable risk factor for all endourological procedures.
- Despite the large number of retrograde intrarenal surgery–related randomized clinical trials (RCTs) and their usage as the most reliable data, there is still no consensus about the comparison of PCS of patients to more accurately claim the full absence of differences between the compared groups.
- Standardized protocols for evaluating and reporting on PCS will improve reporting and comparison while helping clinicians make better treatment choices and informed decision-making with patients.

## Introduction

Retrograde intrarenal surgery (RIRS) is integral to kidney stone treatment. Despite the constant modernization of flexible ureteroscopes and the expansion of their indications, many factors, including operator-related, intervention-related, and patient-related factors, contribute to the success of this procedure.^1^^,^^2^ The latter, in turn, are divided into modifiable and non-modifiable factors, which are not unique to RIRS and underlie medical practice as a whole.^3^ The pelvicalyceal system (PCS) is a highly variable anatomical zone and one of the non-modifiable factors, the analysis of which is fundamental in choosing a surgical strategy and predicting the success of treatment of renal calculi and associated complications.^4^ On the other hand, according to the latest guidelines, RIRS is recommended as a first-line approach when treating ≤2 cm stones, especially those located in the lower pole with unfavorable anatomy for extracorporeal shockwave lithotripsy (ESWL).^5^ Among primary studies, randomized controlled trials (RCTs) are the most reliable and determine the level of evidence for specific statements in clinical recommendations. Randomized controlled trials are based on the randomization of patients to minimize selection biases; therefore, special attention in all original studies is given to comparing various factors between groups.^6^ Despite the importance of this step, there is no attempt to analyze the methodology of RIRS-related RCTs, namely the comparison of patients’ PCS anatomy, before stating the absence of any differences between patients. Considering the above, the purpose of this methodological review is to analyze available RCTs comparing RIRS with other treatment modalities, to determine the extent of comparison of PCS anatomy between patients.

## Materials and Methods

### Search

In December 2024, a search was conducted in databases including PubMed, Google Scholar, Scopus, and clinicaltrials.gov using Boolean operators and the following search terms: “retrograde intrarenal surgery,” “RIRS,” “Flexible ureteroscopy,” “fURS,” and “Ureteroscopy.” The search was limited explicitly to publications that describe comparisons of experimental and control groups in the context of RIRS for stones only in the kidney (PCS).

### Inclusion Criteria

We searched for English-language papers with full article accessibility, randomized studies, descriptions of pre-procedural set-up and preoperative imaging modalities used, and surgery-related metrics (stone-free rate (SFR), operative time, fluoroscopy time, complication rate, retreatment rate, auxiliary procedure rate). To reduce the search volume without losing the representativeness of the results, only RCTs comparing RIRS with other modalities without publication date restriction were included due to their highest level of evidence in the hierarchy of primary research.

### Exclusion Criteria

Non-English written and inaccessible papers, non-RCTs and other study designs, and a lack of information about surgery-related metrics.

### Studies Process

Two reviewers (A.T. and E.M.) independently identified all papers. All studies fitting the inclusion criteria were selected for full review. If there was a disagreement or discrepancy, the senior author (B.K.S.) made the final decision.

### Data Extraction and Analysis

We analyzed the parameters used in the selected studies to compare the differences between the groups, focusing on PCS anatomy. These parameters were taken either from the “Materials and methods” section or related tables and grouped according to the specific characteristics of PCS as a whole or its separate compartments, namely: presence and/or stage of hydronephrosis, pelvic branching, and position (intrarenal, extrarenal) related to the parenchyma, whether a literature classification system was used, the number and orientation of minor calyces, the level of their grouping, and direct measurements (angles, length, width, etc.). The use of specialized nomograms was also analyzed based on the stone’s location in specific PCS. Finally, if there were any indications in the articles that the authors studied the anatomy of the PCS of recruited patients (e.g., exclusion of cases with anomalous kidneys, intraoperative characterization), then this fact was noted in a separate column in the table. The review was reported according to the Preferred Reporting Items for Systematic Reviews and Meta-Analyses (PRISMA) guidelines to ensure transparency and comprehensive examination of the topic.

## Results

**Figure 1.**
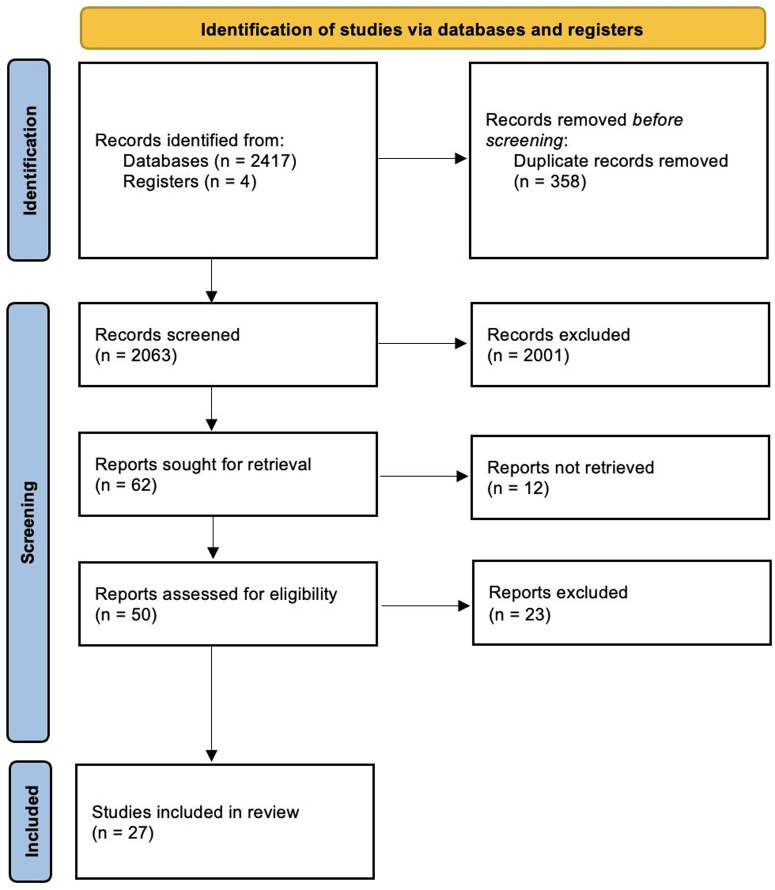
Preferred reporting items for systematic reviews and meta-analyse flow diagram of literature search.

Following the inclusion criteria in the literature search, the final analysis included 27 publications from 2421 articles (Figure 1). Among them, RIRS was compared only with ESWL, only with 1 percutaneous modality, with different percutaneous modalities, with both ESWL and a percutaneous modality, and in combination with other modalities in 5,^9^^,^^12^^,^^14^^,^^15^^,33^ 17,^7^^,^^8^^,^^13^^,^^16^^-^ ^18^^,^^20^^-^ ^23^^,^^25^^,^^27^^-32^ 1,^24^ 3,^11^^,^^19^^,^^26^ and 1^10^ studies, respectively (Table 1). 12 studies were focused on inferior pole calculi.^9^^,^^11^^,^^12^^,^^17^^-^ ^20^^,^^21^^,^^23^^,^^24^^,^^30^^,^^33^ The number of participants in the RIRS arm varied from 11 to 207.^19^^,26^ Pros and cons of RIRS over other modalities were found to be controversial. Operative time was better, non-inferior, or worse than ESWL or different percutaneous approaches in 0/3/3 and 5/10/4 studies, respectively. Fluoroscopy time was better, non-inferior, or worse in 1/0/1 and 8/1/1 studies. SFR rate was better, non-inferior, or worse in 5/3/0 and 1/12/8 studies.

According to the analysis, ultrasound (US), kidney-ureter-bladder (KUB) X-ray, intravenous urography (IVU), non-enhanced and enhanced computed tomography (CT) were preoperatively performed in 7, 6, 7, 21, and 5 studies, respectively (Table 2). Based on them, hydronephrosis presence and/or degree were analyzed in 8 studies. In 13 studies, authors compared stone localization between groups and indicated PCS compartments as pelvis, superior, middle, and inferior poles. Moreover, in 2 studies, authors compared the Seoul National University Renal Stone Complexity (S-reSC) scores between groups, which is also based on stone location in different PCS compartments.^34^ The authors investigated and excluded cases with anomalous kidney anatomy in 18 studies. In 1 study, authors excluded cases with unfavorable lower pole anatomy among patients with stones in this location; however, a direct comparison between the control and experimental groups was missed.^19^ Direct morphometric measurements were compared in 4 studies and were focused on the lower pole only, namely the infundibulopelvic angle (IPA), infundibular length (IL), and infundibular width (IW). Features such as the position of the renal pelvis in relation to the kidney parenchyma (intrarenal or extrarenal), the number and orientation of calyces, and the existing PCS classifications were not compared or used.

## Discussion

Currently, endourological interventions have almost completely replaced open surgery in the treatment of kidney stone disease.^35^ Improved imaging techniques, instruments, and experience within the urological community have undoubtedly facilitated this.^36^ Many factors determine the outcome of the intervention and the reliability of the results obtained, such as sample size of patients,^7^ single-center performance,^14^ variability of equipment used in different centers,^19^ performance by the same operator,^27^ various types and timing of postoperative imaging,^31^ stone composition,^9^ and others. However, the specific characteristics of the patients themselves are also important, which directly influence potential differences between groups and may themselves determine the results obtained.^13^^,^^16^^,^^29^

The anatomy of any organ is a non-modifiable aspect, unlike other potential risk factors for failure during surgery. The importance of the surgical anatomy of the collecting system may favor 1 procedure over others, especially in the context of Mini PCNL and RIRS, when comparing results.^31^ According to this review, 25 studies (92.6%) generally investigated renal anatomy, and the remaining 2 articles assessed the treatment results of staghorn stones or isolated upper pole stones.^10^^,^^32^ In the latter study, the anatomical assessment would be helpful given the presence of upper-pole-based morphometric features and the possibility of visualizing the rest of the non-affected PCS, especially in the case of the presence of pelvic division into 2 or more separate branches.^37^

In most studies, the authors looked at the outcomes by excluding renal anomalies or abnormal renal anatomy (18, 66.7% papers). However, this leads to a slight bias in reporting and comparing studies. One study, when looking at the results of Mini PCNL and RIRS in the management of solitary 1-2 cm stones, found inferior SFR among lower pole calculi and attributed it to intraoperatively defined unfavorable factors like IPA, IW, and IL.^27^ Moreover, the study is 1 of 5 in which the authors preoperatively used contrast-enhanced CT.

Another feature of PCS anatomy is its division into compartments such as the pelvis, upper, middle, and lower poles to describe the location of the stone. Of 14 studies that analyzed endourological outcomes, 11 had this description, but none provided any details about the number and orientation of minor calyces. While this detailed characterization of the PCS could be time-consuming, it would help to understand the outcomes better. Also, considering the various movements and muscles of the wrist and hand, these factors can directly affect the ergonomics and results of RIRS.

Guglielmetti et al^38^ spoke about parameters extracted from CT that could predict renal calyceal access during PCNL, and the only independent predictive factor was the angle between the entrance calyx and the desired calyx. Ricapito et al^39^ stated that anterior and posterior accesses in supine PCNL offer similar safety and efficacy, and access is usually decided by surgeons when investigating PCS anatomy, which confirms subjectivity in this aspect and requires an objective comparison of at least anterior and posterior minor calyces between groups.

Despite the main focus of this review on RIRS, a significant part of the work was compared with percutaneous surgery. A potential solution to this problem was mentioned in 2 works, where the authors used the S-reSC when comparing patient characteristics. This is based on stone location coupled with the orientation of affected calyces using the frontal plane of the kidney.^13^^,^^25^ In turn, the problem of comparing the number of minor calyces is more evident in studies involving patients with multiple stones, especially within various locations. In the literature, controversies also exist on this question. Demirbas et al^40^ concluded that RIRS is a more effective and reliable procedure than PCNL, with higher SFRs and lower complication rates in treating multi-calyceal and multiple stones in the same renal unit. In contrast, Baran and Aykac stated the opposite opinion.^41^ As was shown by several included studies in this review, such a scenario is an independent risk factor for intervention failure.^22^^,^^29^ Despite this, such studies often lack clarification regarding the number and location of multiple stones. Only 1 study indicates different percentages of multiple stones and multiple localizations of stones, which directly confirms the hypothesis of the possibility of including patients with the same stone burden but different complexity in a single group.^14^

The next most common anatomical sign is the presence and/or grade of hydronephrosis. Wang et al^42^ showed that in the presence of severe hydronephrosis, the postoperative urinary sepsis rate in the RIRS group was as high as 15.4%, which was much higher than the 1.5% rate observed in the S-PCNL group. Ergani et al^44^ concluded that the success of FURS will decrease as the grade of hydronephrosis increases to grade 2 or more, whereas Özman et al^43^ reported the opposite results. These contradictions make it difficult to rule out the impact of hydronephrosis on endourological outcomes definitively. It seems prudent, therefore, to analyze this in future studies to clearly understand its effect on the results of RIRS outcomes. Among the analyzed studies, only 8 (29.6%) are provided with a comparison of hydronephrosis between groups, while 5 of these relate to studies related to lower pole calculi (41.7%). In an overwhelming 96.3% of studies (26/27), a pre-operative CT scan was used to determine the hydronephrosis grade. One study was conducted using US and IVU.^7^ Also, some studies argue that CT are not always needed for PCS anatomy, while others argue that they are helpful for anatomy and predicting outcomes.^45^^,^^46^

In addition to the qualitative characteristics mentioned earlier, there are also quantitative parameters for assessing PCS based on determining the IPA, IL, and IW. Only 4 of the included studies, all focusing on lower pole calculi, provided such a comparison between groups. However, the remaining 8 out of 12 studies (66.7%) did not include these features. Some studies acknowledged the lack of this data or correlation analysis.^9^^,^^23^ Another study used specific cut-offs for IPA, under 45 degrees, when comparing RIRS and ESWL for lower pole stones between 1 and 2 cm.^33^ However, they did not investigate IL and IW, which affected outcomes. Bozzini et al^19^ excluded cases with unfavorable lower pole anatomy (IPA <30°, IL >10 mm, and IW<5 mm), leaving their influence on results uncertain.

Some authors directly cite steep IPA for converting RIRS to percutaneous surgery. However, they do not have any direct measurements and are based only on their subjective opinion.^8^^,^^20^ According to other studies, the SFR following RIRS for managing lower pole calculi was worse than ultra-mini PCNL and mini PCNL.^16^^,^^27^ However, these studies did not compare anatomical factors and grade of hydronephrosis. Moreover, the RIRS group contained significantly more patients with multiple stones in 1 study.^16^

A recent meta-analysis demonstrated that IPA is an independent factor influencing the success of RIRS.^47^ However, the authors state that high heterogeneity characterizes the analyzed studies, partly due to different measurement approaches. All of the studies in this review that compare IPA between patients or use it to exclude patients with steep angles lack information regarding the measurement method used. Notably, these morphometric measurements extend beyond the lower poles, enabling the determination of angles between various calyceal levels. As was shown by Aminsharifi et al,^48^ significant hydronephrosis coupled with an upper calyx-lower calyx infundibular angle was associated with a greater likelihood of stone scattering, which could potentially affect the outcome of PCNL.

Finally, none of the studies used the different classifications of PCS based on its branching or division site related to the kidney parenchyma. Literature suggests that these factors are of prognostic significance. According to the Sampaio and Mandarim-De-Lacerda nomenclature,^49^ systems with crossed calyces draining the kidney midzone (type A2) showed lower accessibility to minor calyces during flexible ureteroscopy.^50^ Kirecci et al^51^ drew similar conclusions, finding that after RIRS, SFR was significantly lower in subgroup A2 (30.4%) and considerably higher in the subgroup with independent drainage of midzone calyces to the pelvis (type B2). Type A2 type also exhibited increased operative and fluoroscopy time.

To the authors’ knowledge, this is the first review to analyze PCS-related anatomical factors in randomized studies that compare RIRS with other endourological modalities. Most studies frequently overlook independent risk factors such as the anatomy of the PCS. While some exclude cases with abnormal anatomy and stone location, studies also missed the degree of hydronephrosis and morphometric measurements. Aspects such as pelvic division and branching, calyceal number, and orientation were missed despite data in the literature confirming their influence on RIRS outcomes. Among studies on lower pole calculi, only 4 of 12 compared the anatomy. Moreover, some authors use these parameters to exclude patients with unfavorable anatomy or associate it with conversion from RIRS to percutaneous surgery but fail to give objective measurements.

Future studies comparing or presenting data on outcomes of RIRS should use a standardized protocol for assessing PCS anatomy to highlight risk factors for surgical failure and reduce heterogeneity in the reporting of studies, as well as when conducting meta-analyses and updating the guidelines.

However, this review also has several limitations that should be mentioned. First, strict inclusion criteria were established, potentially leading to the omission of similar RCTs. Secondly, randomized studies were included regardless of blinding, which could affect study quality. Finally, other patient and stone-related features may also be varied among studies but were not analyzed here and should be investigated in future studies.

This review shows gaps in the literature while assessing and reporting on PCS anatomy in studies with RIRS. Essential factors such as pelvic division, branching patterns, and calyceal orientation are broadly not mentioned. Despite influencing procedural outcomes, the presence and degree of hydronephrosis and morphometric measurements are often missed. Unless studies mention these anatomical factors without excluding certain groups of patients, it is difficult to compare outcomes between modalities and in between studies. Standardized protocols for evaluating and reporting on PCS will improve reporting and comparison while helping clinicians make better treatment choices and informed decision-making with patients.

## Figures and Tables

**Table 1.** Randomized Clinical Trials Comparing Retrograde Intrarenal Surgery with Other Modalities

No.	Authors	Purpose	Year	Participants (n)	Anatomy-Related Limitations of Study	RIRS Was Better In	RIRS Was Worse In	No Difference In Or Significance Is Not Calculated	
1	Bryniarski et al^7^	To compare results of PCNL and RIRS in the management of >2 cm renal stones	2012	35 vs 35	none	- operative time	- SFR	none	
2	Sabnis et al^8^	To compare results of microperc and RIRS in the management of small renal calculi	2013	35 vs 35	none	none	none	- operative time - complications rate - auxiliary procedures rate - SFR	
3	Singh et al^9^	To compare RIRS and ESWL in the management of intermediate size inferior pole calculi	2014	35 vs 35	-no consideration of inferior pole anatomy	- SFR, - retreatment rate - auxiliary procedures rate	- operative time	- complications rate	
4	Zhong et al^10^	To compare PCNL+miniPCNL and PCNL+ RIRS in the management of staghorn calculi in solitary kidney	2014	23 vs 22	none	- operative time - SFR - total tracts number		- complication rate - auxiliary procedures rate	
5	Kumar et al^11^	To compare ESWL, RIRS and miniPCNL in the management of 10-20 mm radiolucent inferior pole calculi	2014	42 vs 43 vs 41	none	- fluoroscopy time (compared to miniPCNL) - retreatment rate - auxiliary procedure (compared to ESWL) - SFR (compared to ESWL)	- SFR (compared to miniPCNL) - auxiliary procedure (compared to miniPCNL)	- operative time - complication rate	
6	Kumar et al^12^	To compare ESWL and RIRS in the management of inferior pole ≤2 cm stones	2015	90 vs 90	none	- retreatment rate	none	- operative time - auxiliary procedures rate - SFR - complications rate	
7	Lee et al^13^	To compare miniPCNL and RIRS in the management of renal stones >10 mm	2015	35 vs 33	none	- auxiliary procedures rate	None	- operative time - SFR - complications rate	
8	Xu et al^14^	To compare of RIRS combined with holmium laser lithotripsy and ESWL in the management of residual calculi after PCNL	2015	46 vs 48	none	- SFR	-none	- complications rate	
10	Javanmard et al^15^	To compare RIRS and ESWL in the management of the Renal Stones ≤2 cm	2016	60 vs 60	none	- retreatment rate -SFR (for lower calculi) - complications rate (steinstrasse)	- operative time	- SFR (for others)	
11	Demirbas et al^16^	To compare ultraminiPCNL and RIRS in the management of 10-25 mm kidney stones	2016	30 vs 43	- different stones localization between groups	- fluoroscopy time	- SFR (for inferior pole calculi)	-operative time -SFR (for others) -complications rate	
	Fayad et al^17^	To compare miniPCNL and RIRS in the management of ≤2 cm inferior pole renal calculi	2016	60 vs 60	none	none	- operative time	-SFR -complications rate	
12	Kandemir et al^18^	To compare microperc and RIRS in the management of inferior pole renal stones ≤15 mm	2017	30 vs 30	none	-fluoroscopy time	none	-operative time -SFR -complications rate	
13	Bozzini et al^19^	To compare ESWL, RIRS, and PCNL in the management of inferior pole stones ≤2cm	2017	194 vs 207 vs 181	- exclusion of cases with challenging patients that would have clearly better results with PCNL	-fluoroscopy time -retreatment rate (compared to ESWL) - auxiliary procedures rate (compared to ESWL) - SFR (compared to ESWL)	none	-operative time	
14	Zeng et al^20^	To compare super-miniPCNL and RIRS in the management of 1-2 cm inferior pole renal calculi	2018	80 vs 80	- most participants are from Asia	none	-SFR -auxiliary procedures rate	-operative time -complications rate	
15	Jiang et al^21^	To compare “all-seeing needle” (micro-PCNL and RIRS flexible ureterorenoscopy (FURS) in the management of lower calyceal stones of ≤2 cm	2018	58 vs 58	none	none	-operative time	-SFR -complications rate -Auxiliary procedures rate	
16	Gucuk et al^22^	To analyze the influence of stone density and location on miniPCNL and RIRS outcomes	2019	30 vs 30	none	-fluoroscopy time	none	-operative time -complications rate -SFR	
17	Jin et al^23^	To compare RIRS and miniPCNL in the management of 1-2 cm inferior pole calculi	2019	110 vs 110	yes (absence of subgroup analysis based on IPA, IL and IW	-complication rate	none		-operative time -SFR
18	Yavuz et al^24^	To compare RIRS, microperc, ultraminiPCNL, miniPCNL, and PCNL in the management of inferior pole renal calculi 1-2 cm	2020	33 vs 35 vs 33 vs 34 vs 33	none	-operative time -fluoroscopy time	-SFR	-retreatment rate -complication rate	
19	Çakıcı et al^25^	To compare RIRS and PCNL in the management of 2-4 cm Kidney Stones	2020	50 vs 50	none	-operative time -fluoroscopy time	-SFR	-complications rate	
20	McCahy et al^26^	To compare ESWL, RIRS and PCNL in the management of 1-2 cm renal stones	2020	10 vs 11 vs 10	none	none	none	- SFR - complications rate - auxiliary procedures rate	
21	Jain et al^27^	To compare miniPCNL and RIRS in the management of solitary renal stone of 1-2 cm	2021	40 vs 40	none	-fluoroscopy time	-operative time -SFR (for lower pole)	-SFR (general) -complications rate -auxiliary procedure rate	
22	Sebaey et al^28^	To compare RIRS and miniPCNL in the management of renal stones 20-30 mm	2022	35 vs 35	none	-fluoroscopy time	-operative time	-complications rate -SFR	
23	Darwish et al^29^	To compare RIRS and miniPCNL in the management of < 2 cm renal calculi	2023	59 vs 59	none	-complications rate	-auxiliary procedure	-SFR -operative time	
24	Rehman et al^30^	To compare miniPCNL and RIRS in the management of inferior pole calculi	2023	75 vs 75	none	none	-SFR	- complications rate	
25	Dutta et al^31^	To compare miniPCNL and RIRS in the management of 1-2 cm renal calculi	2023	51 vs 50	none	- fluoroscopy time	-SFR	- operative time - complications rate - retreatment rate	
26	Assem et al^32^	To compare supracostal ultrasound guided PCNL (SUGA-PCNL) and RIRS In the management of large isolated upper calyceal stones	2024	42 vs 41	none	- operative time	-SFR	- fluoroscopy time - retreatment rate	
27	Abd Elal et al^33^	To compare RIRS and ESWL in the management of 1-2 cm radiolucent inferior pole renal calculi	2024	RIRS 45 vs SWL 47	- exclusion of cases with IPA less 45 degree	- SFR - retreatment rate	- operative time - Fluoroscopy time	- complications rate- auxiliary procedure rate	

ESWL, extracorporeal shock wave lithotripsy; IPA, infundibulopelvic angle; IL, infundibular length; IW, infundibular width; PCNL, percutaneous nephrolithotripsy; RIRS, retrograde intrarenal surgery; SFR, stone-free rate; SUGA, supracostal ultrasound guided.

**Table 2.** Pelvicalyceal System–Related -Factors Compared Between Groups in Selected Studies

No.	Authors	Imaging Used in the Study	Whether Authors Investigated Kidney Anatomy?	Hydronephrosis	Pelvical Position and Division	Existing Classifications	Calyceal Grouping	Number and Orientation of Calyces	Direct Measurements	Related Nomograms
1	Bryniarski et al^7^	US, IVU	Yes (anomaly excluded)	Yes (presence)	–	–	–	–	–	–
2	Sabnis et al^8^	NECT	Yes	–	–	–	Yes	–	–	–
3	Singh et al^9^	US, X-ray KUB, IVU, NECT	Yes (anomaly excluded)	–	–	–	–	–	–	–
4	Zhong et al^10^	X-ray KUB, IVU, NECT	None	–	–	–	–	–	–	–
5	Kumar et al^11^	US, X-ray KUB, NECT	Yes (anomaly excluded)	–	–	–	–	–	–	–
6	Kumar et al^12^	US, X-ray KUB, NECT	Yes	Yes (grade)	–	–	–	–	IPA IL IW	–
7	Lee et al^13^	NECT	Yes (anomaly excluded)	Yes (presence)	–	–	Yes	–	–	S-ReSC score
8	Xu et al^14^	CECT	Yes (anomaly excluded)	–	–	–	Yes	–	–	–
10	Javanmard et al^15^	CECT	Yes (anomaly excluded)	–	–	–	Yes	–	–	–
11	Demirbas et al^16^	IVU, low-dose NECT	Yes (anomaly excluded)	–	–	–	Yes	–	–	–
	Fayad et al^17^	KUB X-ray, NECT	Yes (anomaly excluded)	–	–	–	–	–	–	–
12	Kandemir et al^18^	NECT	Yes (anomaly excluded)	Yes (grade)	–	–	–	–	–	–
13	Bozzini et al^19^	CECT	Yes (exclusion of cases with IPA <30, IL>10 mm and IW<5 mm)	–	–	–	–	–	–	–
14	Zeng et al^20^	IVU, NECT	Yes (anomaly excluded)	Yes (grade)	–	–	–	–	- IPA - IL - IW	–
15	Jiang et al^21^	US, IVU, NECT	Yes (anomaly excluded)	Yes (grade)	–	–	–	–	–	–
16	Gucuk et al^22^	NECT	Yes (anomaly excluded)	–	–	–	Yes	–	–	–
17	Jin et al^23^	NECT	Yes (anomaly excluded) -Severe hydronephrosis excluded	Yes (grade)	–	–	–	–	- IPA - IL - IW	–
18	Yavuz et al^24^	US, NECT	Yes (anomalous kidneys are excluded)	–	–	–	–	–	–	–
19	Çakıcı et al^25^	NECT	Yes	–	–	–	–	–	–	S-ReSC score
20	McCahy et al^26^	NECT	Yes	–	–	–	Yes	–	–	–
21	Jain et al^27^	CECT	Yes (subjectively analyzed anatomy during RIRS)	–	–	–	Yes	–	–	–
22	Sebaey et al^28^	NECT	Yes (anomalous kidneys are excluded)	–	–	–	Yes	–	–	–
23	Darwish et al^29^	NECT	Yes	Yes (grade)	–	–	Yes	–	–	–
24	Rehman et al^30^	US, NECT	Yes (anomalous kidneys are excluded)	–	–	–	–	–	–	–
25	Dutta et al^31^	NECT	Yes (anomalous kidneys are excluded)	–	–	–	Yes	–	–	–
26	Assem et al^32^	CECT	None	–	–	–	–	–	–	–
27	Abd Elal et al^33^	US, IVU, X-ray KUB, NECT	Yes (anomalous kidneys are excluded)	–	–	–	–	–	- IPA	–

CECT, contrast-enhanced computed tomography; IL, infundibular length; IPA, infundibulopelvic angle; IVU, intravenous urography; IW, infundibular width; NECT, non-enhanced computed tomography; US, ultrasound; X-ray KUB, kidney, ureter and bladder X-ray.

## Data Availability

The data that support the findings of this study are available on request from the corresponding author.

## References

[1] Akgül M Çakır H Çinar Ö The efficacy and safety of retrograde intrarenal surgery: a multi-center experience of the RIRSearch group study. J Urol Surg. 2023;10(2):119 128. (doi: 10.4274/jus.galenos.2023.2022.0039)

[2] Silva THCD Passerotti CC Pontes Júnior J Maximiano LF Otoch JP Cruz JASD. The learning curve for retrograde intrarenal surgery: a prospective analysis. Rev Col Bras Cir. 2022;49:e20223264. (doi: 10.1590/0100-6991e-20223264-en) PMC10578857 35946637

[3] Alwan NA Stannard S Berrington A Risk factors for ill health: how do we specify what is ‘modifiable’? PLoS Glob Public Health. 2024;4(3):e0002887. (doi: 10.1371/journal.pgph.0002887) PMC10911600 38437177

[4] Karim SS Hanna L Geraghty R Somani BK. Role of pelvicalyceal anatomy in the outcomes of retrograde intrarenal surgery (RIRS) for lower pole stones: outcomes with a systematic review of literature. Urolithiasis. 2020;48(3):263 270. (doi: 10.1007/s00240-019-01150-0) 31372691 PMC7220875

[5] Geraghty RM Davis NF Tzelves L Best practice in interventional management of urolithiasis: an update from the European Association of Urology guidelines panel for urolithiasis 2022. Eur Urol Focus. 2023;9(1):199 208. (doi: 10.1016/j.euf.2022.06.014) 35927160

[6] Kahan BC Rehal S Cro S. Risk of selection bias in randomised trials. Trials. 2015;16(1):405. (doi: 10.1186/s13063-015-0920-x) PMC4566301 26357929

[7] Bryniarski P Paradysz A Zyczkowski M Kupilas A Nowakowski K Bogacki R. A randomized controlled study to analyze the safety and efficacy of percutaneous nephrolithotripsy and retrograde intrarenal surgery in the management of renal stones more than 2 cm in diameter. J Endourol. 2012;26(1):52 57. (doi: 10.1089/end.2011.0235) 22003819

[8] Sabnis RB Ganesamoni R Doshi A Ganpule AP Jagtap J Desai MR. Micropercutaneous nephrolithotomy (microperc) vs retrograde intrarenal surgery for the management of small renal calculi: a randomized controlled trial. BJU Int. 2013;112(3):355 361. (doi: 10.1111/bju.12164) 23826843

[9] Singh BP Prakash J Sankhwar SN Retrograde intrarenal surgery vs extracorporeal shock wave lithotripsy for intermediate size inferior pole calculi: a prospective assessment of objective and subjective outcomes. Urology. 2014;83(5):1016 1022. (doi: 10.1016/j.urology.2013.12.026) 24560970

[10] Zhong W Zhao Z Wang L Swami S Zeng G. Percutaneous-based management of staghorn calculi in solitary kidney: combined mini percutaneous nephrolithotomy versus retrograde intrarenal surgery. Urol Int. 2015;94(1):70 73. (doi: 10.1159/000360708) 25034200

[11] Kumar A Kumar N Vasudeva P Kumar Jha S Kumar R Singh H. A prospective, randomized comparison of shock wave lithotripsy, retrograde intrarenal surgery and miniperc for treatment of 1 to 2 cm radiolucent lower calyceal renal calculi: a single center experience. J Urol. 2015;193(1):160 164. (doi: 10.1016/j.juro.2014.07.088) 25066869

[12] Kumar A Vasudeva P Nanda B Kumar N Das MK Jha SK. A prospective randomized comparison between shock wave lithotripsy and flexible ureterorenoscopy for lower caliceal stones ≤2 cm: a single-center experience. J Endourol. 2015;29(5):575 579. (doi: 10.1089/end.2013.0473) 25203489

[13] Lee JW Park J Lee SB Son H Cho SY Jeong H. Mini-percutaneous Nephrolithotomy vs Retrograde intrarenal Surgery for Renal Stones Larger Than 10 mm: a Prospective randomized controlled trial. Urology. 2015;86(5):873 877. (doi: 10.1016/j.urology.2015.08.011) 26320082

[14] Xu G Wen J Li Z A comparative study to analyze the efficacy and safety of flexible ureteroscopy combined with holmium laser lithotripsy for residual calculi after percutaneous nephrolithotripsy. Int J Clin Exp Med. 2015;8(3):4501 4507. 26064375 PMC4443209

[15] Javanmard B Kashi AH Mazloomfard MM Ansari Jafari A Arefanian S. Retrograde intrarenal Surgery versus shock wave lithotripsy for Renal Stones Smaller Than 2 cm: a Randomized Clinical Trial. Urol J. 2016;13(5):2823 2828. 27734422

[16] Demirbas A Resorlu B Sunay MM Karakan T Karagöz MA Doluoglu OG. Which should be preferred for moderate-size kidney stones? Ultramini Percutaneous Nephrolithotomy or Retrograde Intrarenal Surgery? J Endourol. 2016;30(12):1285 1289. (doi: 10.1089/end.2016.0370) 27706948

[17] Fayad AS Elsheikh MG Ghoneima W. Tubeless mini-percutaneous nephrolithotomy versus retrograde intrarenal surgery for lower calyceal stones of ≤2 cm: a prospective randomised controlled study. Arab J Urol. 2017;15(1):36 41. (doi: 10.1016/j.aju.2016.10.002) 28275516 PMC5329753

[18] Kandemir A Guven S Balasar M Sonmez MG Taskapu H Gurbuz R. A prospective randomized comparison of micropercutaneous nephrolithotomy (Microperc) and retrograde intrarenal surgery (RIRS) for the management of lower pole kidney stones. World J Urol. 2017;35(11):1771 1776. (doi: 10.1007/s00345-017-2058-9) 28589217

[19] Bozzini G Verze P Arcaniolo D A prospective randomized comparison among SWL, PCNL and RIRS for lower calyceal stones less than 2 cm: a multicenter experience: a better understanding on the treatment options for lower pole stones. World J Urol. 2017;35(12):1967 1975. (doi: 10.1007/s00345-017-2084-7) 28875295

[20] Zeng G Zhang T Agrawal M Super-mini percutaneous nephrolithotomy (SMP) vs retrograde intrarenal surgery for the treatment of 1-2 cm lower-pole renal calculi: an international multicentre randomised controlled trial. BJU Int. 2018;122(6):1034 1040. (doi: 10.1111/bju.14427) 29873874

[21] Jiang K Chen H Yu X Chen Z Ye Z Yuan H. The “all-seeing needle” micro-PCNL versus flexible ureterorenoscopy for lower calyceal stones of ≤ 2 cm. Urolithiasis. 2019;47(2):201 206. (doi: 10.1007/s00240-018-1049-7) 29497768

[22] Gucuk A Yilmaz B Gucuk S Uyeturk U. Are stone density and location useful parameters that can determine the endourological surgical technique for kidney stones that are smaller than 2 cm? A prospective randomized controlled trial. Urol J. 2019;16(3):236 241. (doi: 10.22037/uj.v0i0.4280) 30178449

[23] Jin L Yang B Zhou Z Li N. Comparative efficacy on flexible ureteroscopy lithotripsy and miniaturized percutaneous nephrolithotomy for the treatment of medium-sized lower-pole renal calculi. J Endourol. 2019;33(11):914 919. (doi: 10.1089/end.2019.0504) 31596612

[24] Yavuz A Kilinc MF Bayar G. Outcomes of different minimally invasive techniques in lower calyceal stones of 1 to 2 centimeters: a prospective, randomized study. Arch Esp Urol. 2020;73(4):307 315. 32379066

[25] Çakıcı MÇ Karakoyunlu N Sari S Comparison of Retrograde intrarenal Surgery and Percutaneous Nephrolithotomy Used in the Treatment of 2-4 cm Kidney Stones in Terms of Pain and Need for Additional analgesics: a Prospective Randomized Study. J Laparoendosc Adv Surg Tech A. 2020;30(12):1301 1307. (doi: 10.1089/lap.2020.0179) 32397802

[26] McCahy PJ Hong M Paul E Berman I Shahbaz S. Shock-wave lithotripsy, ureterorenoscopy and percutaneous nephrolithotomy for 1-2 cm renal stones: a randomised pilot study. J Clin Urol. 2020;13(6):413 418. (doi: 10.1177/2051415820935663)

[27] Jain M Manohar CS Nagabhushan M Keshavamurthy R. A comparative study of minimally invasive percutaneous nephrolithotomy and retrograde intrarenal surgery for solitary renal stone of 1-2 cm. Urol Ann. 2021;13(3):226 231. (doi: 10.4103/UA.UA_10_20) 34421256 PMC8343287

[28] Sebaey A Taleb AA Elbashir S Gomaa R Elshazli A Saber W. Flexible ureterorenoscopy (RIRS) vs. mini- percutaneous nephrolithotomy (MINI-PCNL) for renal stones 20-30 mm a prospective randomized study. Afr J Urol. 2022;28(1):1 6.

[29] Darwish AE Abdel Moneim AE Ahmed AI Hamdy SM Abolella HA Reda A. A randomized comparative study of flexible ureterorenoscopy versus mini-percutaneous nephrolithotomy for treatment of renal stones 2 cm or Less. Curr Urol. 2024;18(4):273 277. (doi: 10.1097/CU9.0000000000000215) 40256303 PMC12004952

[30] Ur Rehman O Imran M Rafaqat M Outcomes in lower pole kidney stone management using mini-percutaneous nephrolithotomy compared with retrograde intra renal surgery: a randomized controlled trial. Cureus. 2023;15(2):e35343. (doi: 10.7759/cureus.35343) PMC10039418 36974241

[31] Dutta R Mithal P Klein I Patel M Gutierrez-Aceves J. Outcomes and costs following mini-percutaneous nephrolithotomy or flexible ureteroscopic lithotripsy for 1-2-cm renal stones: data from a prospective, randomized clinical Trial. J Urol. 2023;209(6):1151 1158. (doi: 10.1097/JU.0000000000003397) 37157794

[32] Assem A Abdalla A Elzoheiry M Supracostal ultrasound guided approach percutaneous nephrolithotomy (SUGA-PNL) versus retrograde intrarenal surgery for large volume isolated upper calyceal stones: a prospective randomized analysis. Urolithiasis. 2024;52(1):154. (doi: 10.1007/s00240-024-01637-5) PMC11522052 39470823

[33] Elal AMA Shaher H El-Barky E Ali S Omar RG. Comparative study between retrograde intrarenal surgery and ultrasound- guided shock wave lithotripsy for treatment of 1 to 2 cm radiolucent lower calyceal stones. Urol Ann. 2024;16(3):185 191. (doi: 10.4103/ua.ua_5_24) 39290222 PMC11404710

[34] Jeong CW Jung JW Cha WH Seoul National University renal stone complexity score for predicting stone-free rate after percutaneous nephrolithotomy. PLoS One. 2013;8(6):e65888. (doi: 10.1371/journal.pone.0065888) PMC3688830 23824752

[35] Tzelves L Geraghty RM Hughes T Juliebø-Jones P Somani BK. Innovations in kidney stone removal. Res Rep Urol. 2023;15:131 139. (doi: 10.2147/RRU.S386844) 37069942 PMC10105588

[36] Talyshinskii A Hameed BMZ Naik N Being all-seeing gymnast within kidney cavity: analysis of the optical and flexibility characteristics trends of 61 flexible ureteroscopes over four decades. Urolithiasis. 2024;52(1):92. (doi: 10.1007/s00240-024-01591-2) 38884642

[37] Kawase K Hamamoto S Taguchi K Impact of pelvicalyceal anatomical variation on surgical outcomes of endoscopic combined intrarenal surgery. BJUI Compass. 2023;4(2):173 180. (doi: 10.1002/bco2.209) 36816147 PMC9931538

[38] Guglielmetti GB Danilovic A Torricelli FCM Coelho RF Mazzucchi E Srougi M. Predicting calyceal access for percutaneous nephrolithotomy with computed tomography multiplanar reconstruction. Clinics (Sao Paulo). 2013;68(6):892 895. (doi: 10.6061/clinics/2013(06)27) 23778484 PMC3674302

[39] Ricapito A Gupta K Savin Z Comparative analysis of safety and efficacy between anterior and posterior calyceal entry in supine percutaneous nephrolithotomy. J Endourol. 2025;39(1):19 25. (doi: 10.1089/end.2024.0508) 39469771

[40] Demirbas A Yazar VM Ersoy E Comparision of percutaneous nephrolithotomy and retrograde intrarenal surgery for the treatment of Multicalyceal and multiple renal stones. Urol J. 2018;15(6):318 322. (doi: 10.22037/uj.v0i0.4213) 29900522

[41] Baran O Aykac A. Comparison of retrograde intrarenal surgery and percutaneous nephrolithotomy in multiple calyceal stones: a match pair analysis of 190 cases. Arch Esp Urol. 2021;74(2):247 253. 33650540

[42] Wang F Hong Y Yang Z Ye L. Comparison of retrograde intrarenal surgery and standard percutaneous nephrolithotomy for management of stones at ureteropelvic junction with high-grade hydronephrosis. Sci Rep. 2021;11(1):14050. (doi: 10.1038/s41598-021-93551-8) PMC8263717 34234219

[43] Özman O Çınar Ö Çakır H Is it a good strategy to proceed a retrograde intrarenal surgery session sheathless after ureteral access sheath insertion failure? A RIRSearch study. J Endourol. 2023;37(7):747 752. (doi: 10.1089/end.2022.0599) 37021344

[44] Ergani B Ozbilen MH Yalcın MY Boyacıoglu H Ilbey YO. The effect of hydronephrosis grade on stone-free rate in retrograde intrarenal stone surgery with flexible ureterorenoscopy. Am J Clin Exp Urol. 2021;9(2):194 201. 34079853 PMC8165711

[45] Rachid Filho D Favorito LA Costa WS Sampaio FJB. Kidney lower pole pelvicaliceal anatomy: comparative analysis between intravenous urogram and three-dimensional helical computed tomography. J Endourol. 2009;23(12):2035 2040. (doi: 10.1089/end.2009.0262) 19916750

[46] Binbay M Akman T Ozgor F Does pelvicaliceal system anatomy affect success of percutaneous nephrolithotomy? Urology. 2011;78(4):733 737. (doi: 10.1016/j.urology.2011.03.058) 21676442

[47] Leighton J Dingwall A Whitehead S Effect of infundibulopelvic angle on outcomes of ureteroscopy: a systematic review and meta-analysis. World J Urol. 2024;42(1):413. (doi: 10.1007/s00345-024-05104-z) PMC11252207 39012390

[48] Aminsharifi A Eslahi A Safarpour AR Mehrabi S. Stone scattering during percutaneous nephrolithotomy: role of renal anatomical characteristics. Urolithiasis. 2014;42(5):435 439. (doi: 10.1007/s00240-014-0678-8) 25026926

[49] Sampaio FJB Mandarim-De-Lacerda CA. Anatomic classification of the kidney collecting system for endourologic procedures. J Endourol. 1988;2(3):247 251. (doi: 10.1089/end.1988.2.247)

[50] Marroig B Frota R Fortes MA Sampaio FJ Favorito LA. Influence of the renal lower pole anatomy and mid-renal-zone classification in successful approach to the calices during flexible ureteroscopy. Surg Radiol Anat. 2016;38(3):293 297. (doi: 10.1007/s00276-015-1562-0) 26438274

[51] Kirecci SL Ilgi M Yesildal C Yavuzsan AH Albayrak AT Sarica K. The impact of the pelvicalyceal anatomy characteristics on the prediction of flexible ureteroscopy outcomes. Urol Ann. 2021;13(2):105 110. (doi: 10.4103/UA.UA_19_20) 34194134 PMC8210722

